# Endoscopic Ultrasound-Guided Radiofrequency Ablation for Pancreatic Metastases: Safety and Clinical Outcomes in a Single-Center Case Series

**DOI:** 10.3390/jcm15155773

**Published:** 2026-07-23

**Authors:** Katarzyna M. Pawlak, Mateusz Jagielski, Aleksander Skórzewski, Eryk Bella, Patryk Kaczor, Jacek Piątkowski, Marek Jackowski

**Affiliations:** Department of General, Gastroenterological and Oncological Surgery, Collegium Medicum Nicolaus Copernicus University, 87-100 Toruń, Poland; matjagiel@gmail.com (M.J.); olek139@gmail.com (A.S.); bella.eryk@gmail.com (E.B.); patryk.kaczor97@gmail.com (P.K.); jpiatkowski@wp.pl (J.P.); jackowscy@hotmail.com (M.J.)

**Keywords:** endoscopic ultrasound, radiofrequency ablation, pancreatic metastases, renal cell carcinoma, local tumor ablation

## Abstract

**Background/Objectives**: Pancreatic metastases are uncommon, and local treatment is reserved for selected patients with limited disease. Surgery remains the standard option when feasible, but may be inappropriate in patients with small, multifocal, anatomically challenging, or medically high-risk lesions. This study evaluated endoscopic ultrasound-guided radiofrequency ablation (EUS-RFA) as an organ-preserving local treatment for pancreatic metastases. **Methods**: Consecutive adult patients undergoing EUS-RFA for histologically confirmed pancreatic metastases at a tertiary endoscopy center between February 2021 and December 2025 were included. All cases were discussed in a multidisciplinary setting. EUS-RFA was performed using a 19G internally cooled RFA needle under real-time EUS guidance. The primary endpoint was initial complete radiological response, defined as complete ablation on first follow-up imaging. Secondary endpoints included technical success, radiological response, repeat EUS-RFA, adverse events, follow-up, and survival. **Results**: Eleven patients were treated; the mean age was 72.4 ± 6.4 years, and 7/11 (63.6%) were women. Primary tumors included renal cell carcinoma in 7/11 (63.6%), breast cancer in 2/11 (18.2%), melanoma in 1/11 (9.1%), and colorectal cancer in 1/11 (9.1%). Median lesion size was 10.5 mm. Technical success was achieved in 11/11 patients (100%). Initial complete radiological response at first follow-up was observed in 10/11 patients (90.9%); the remaining patient achieved complete response after repeat EUS-RFA. Adverse events occurred in 3/11 patients (27.3%), all mild and conservatively managed. No bleeding, perforation, pancreatic duct stenosis, pancreatic collection, or procedure-related death occurred. Median follow-up was 338 days. **Conclusions**: EUS-RFA appears feasible and generally well tolerated in carefully selected patients with pancreatic metastases, particularly small, well-visualized lesions when surgery is not feasible or an organ-preserving strategy is preferred. Larger prospective multicenter studies are needed. Although formal subgroup comparisons were not possible in this small cohort, lesion size, anatomical location, and primary tumor biology are likely to influence technical difficulty, local response, and the need for staged or repeat treatment.

## 1. Introduction

Endoscopic ultrasound-guided radiofrequency ablation (EUS-RFA) has emerged as a minimally invasive therapeutic option for selected pancreatic lesions. By combining high-resolution endosonographic imaging with real-time needle-guided delivery of thermal energy, EUS-RFA enables local tumor ablation while potentially reducing the morbidity associated with pancreatic surgery. To date, most clinical experience has been reported in pancreatic neuroendocrine neoplasms, where EUS-RFA has shown encouraging feasibility, efficacy, and safety in carefully selected patients [1,2]. More recently, its potential role has been explored in other pancreatic tumors, including locally advanced pancreatic cancer and metastatic lesions, although the evidence remains limited [1,2].

Metastases to the pancreas are uncommon and represent a small proportion of pancreatic malignancies. Among secondary pancreatic tumors, renal cell carcinoma is the most frequent primary malignancy, followed by other tumors such as lung cancer, colorectal cancer, melanoma, breast cancer, and sarcoma [3,4]. Pancreatic metastases may occur years after treatment of the primary tumor and can present as isolated, oligometastatic, or as part of disseminated disease. In selected patients, especially those with limited pancreatic involvement and controlled extra-pancreatic disease, local treatment may contribute to disease control and delay the need for systemic treatment escalation [3,4].

Surgical resection remains the standard local treatment for selected patients with resectable pancreatic metastases. However, only a minority of patients present with a single, potentially resectable pancreatic lesion [3]. The benefit of metastasectomy in terms of patient survival has been most consistently observed for metastases from renal cell carcinoma, whereas for other primary tumors, the role of surgery remains often mainly palliative [4]. Moreover, pancreatic surgery is technically demanding and may be associated with substantial morbidity, particularly in patients with multiple lesions, lesions located in different pancreatic segments, previous abdominal surgery, comorbidities, or limited functional reserve. Therefore, pancreatic metastasectomy should be considered only after careful multidisciplinary assessment, balancing potential oncological benefit against procedural risk [3,4].

In this context, and particularly when surgery cannot be performed for medical, anatomical, oncological, or patient-related reasons, EUS-RFA may represent an attractive organ-preserving alternative or complementary local treatment. This approach is particularly appealing for small pancreatic metastases, multifocal disease, patients who are poor surgical candidates, or situations in which repeated pancreatic resections would be undesirable. Early data on EUS-RFA for pancreatic metastases, particularly from renal cell carcinoma, are encouraging, showing high technical success and potential local tumor control [5,6,7]. In the prospective study by Chanez et al., EUS-RFA for pancreatic metastases from RCC was feasible and associated with promising local control [5]. More recently, Stouvenot et al. reported technical success in all treated lesions, with complete or partial radiological response in a substantial proportion of patients, although repeat procedures and adverse events were observed [6].

Several clinically relevant questions remain unresolved, including optimal patient selection, technical settings, number of ablation sessions, radiological criteria for treatment response, durability of local control, and the safety profile of EUS-RFA in metastatic pancreatic lesions. Moreover, metastatic tumors may differ biologically and technically from primary pancreatic lesions, potentially requiring different ablation strategies or repeated treatment sessions to achieve adequate local control.

The aim of the present study was therefore to evaluate the effectiveness and safety of EUS-guided RFA for pancreatic metastases. We hypothesized that EUS-RFA may provide a feasible and safe minimally invasive local treatment option in selected patients with metastatic lesions to the pancreas, particularly when surgery is not feasible, carries excessive risk, or when an organ-preserving approach is preferred.

## 2. Methods

### 2.1. Study Design and Setting

This study included consecutive patients who underwent endoscopic ultrasound-guided radiofrequency ablation (EUS-RFA) for metastatic lesions to the pancreas. We enrolled all patients treated at the tertiary Endoscopy Center, Department of General, Gastroenterological and Oncological Surgery, Collegium Medicum, Nicolaus Copernicus University in Toruń, Poland, between from 9 February 2021, to 3 December 2025, in whom initial EUS-guided assessment confirmed the presence of pancreatic metastases suitable for local ablative treatment. The data were retrospectively assessed.

All lesions had been previously confirmed by EUS-guided tissue acquisition. Each case was subsequently discussed during a multidisciplinary meeting, together with the patient, in order to determine the optimal individualized treatment strategy. EUS-RFA was considered when local therapy was clinically indicated and surgery was not feasible, carried excessive risk, or was not accepted by the patient.

The study was approved by the Ethics Committee at the Collegium Medicum of the Nicolaus Copernicus University (KB 379/2023/6/10) and was conducted in accordance with the Declaration of Helsinki. All patients provided informed consent for endoscopic procedures.

### 2.2. Patients

Adult patients aged ≥18 years with histologically confirmed metastatic lesions to the pancreas were eligible for inclusion. Metastases could originate from renal cell carcinoma or other primary tumors, provided that the pancreatic lesion was considered suitable for local endoscopic treatment.

Inclusion criteria were: age ≥ 18 years; diagnosis of pancreatic metastasis confirmed by EUS-guided tissue acquisition; lesion size suitable for EUS-RFA, preferably 5–20 mm; up to three pancreatic metastatic lesions considered for treatment; Eastern Cooperative Oncology Group performance status 0–2; controlled or limited extra-pancreatic disease according to multidisciplinary assessment; and ability to undergo follow-up imaging.

Patients were excluded in cases of inability to tolerate deep sedation or general anesthesia, uncorrectable coagulopathy, severe bleeding disorder, severe cardiopulmonary or renal disease precluding endoscopy, lack of safe EUS access to the lesion, previous local treatment of the target lesion, or inability to comply with follow-up.

### 2.3. Pre-Procedural Assessment

Before EUS-RFA, all patients underwent clinical evaluation, laboratory testing, and cross-sectional imaging with contrast-enhanced computed tomography, optionally with magnetic resonance imaging. Tumor characteristics were recorded, including the number of pancreatic metastases, maximum lesion diameter, location within the pancreas, relationship to the main pancreatic duct, proximity to major vessels, enhancement pattern, and presence of extra-pancreatic disease.

**Figure 1:**
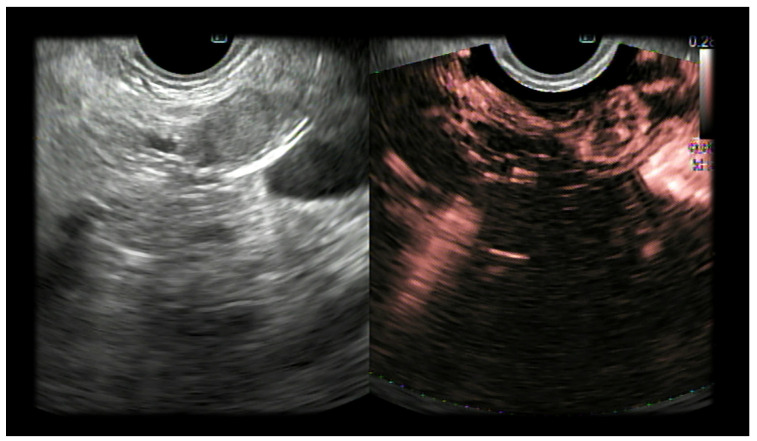
EUS assessment performed prior to EUS-guided RFA, demonstrating typical morphology of RCC pancreatic metastasis with vascular enhancement on DFI (Detective Flow Imaging).

A diagnostic EUS examination was performed before ablation to confirm lesion visibility, assess technical feasibility, evaluate the distance from the main pancreatic duct and vessels, and plan the safest puncture route (Figure 1).

### 2.4. EUS-RFA Procedure

EUS-RFA was performed using a longitudinal echoendoscope (Pentax Medical, Tokyo, Japan). Procedures were performed under deep sedation or general anesthesia, according to local practice and patient-related factors, by two experienced therapeutic endosonographers.

**Figure 2:**
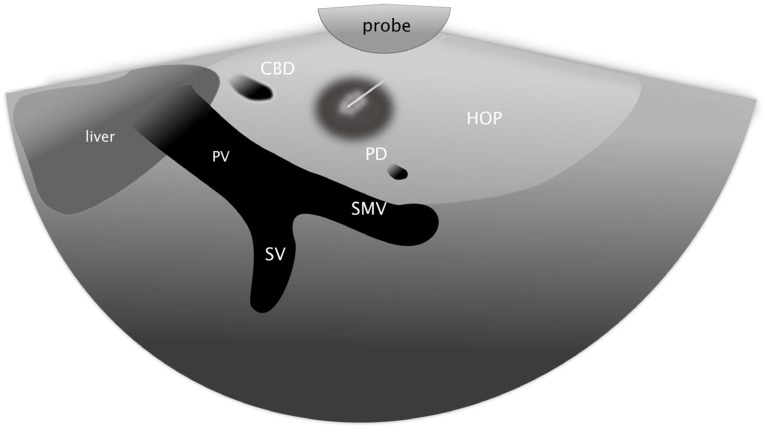
Schematic illustration of the EUS-RFA procedure for pancreatic metastases. A 19G internally cooled RFA needle with a 5–10-mm active tip is advanced through a channel of a longitudinal echoendoscope into the pancreatic metastatic lesion under EUS guidance.

A commercially available EUS-RFA system (EUSRA, STARmed/Taewoong Medical, Gimpo-si, Gyeonggi-do, Republic of Korea) was used. The system consisted of a 19G RFA needle electrode with a 5–10-mm active tip and an internal cooling system connected to an external pump circulating chilled saline during energy delivery. The radiofrequency electrode was introduced into the target lesion under EUS guidance (Figure 2). Doppler imaging was used before puncture to avoid intervening vessels, and the active tip was positioned within the lesion while avoiding the main pancreatic duct, major vessels, and adjacent gastrointestinal wall whenever possible.

Radiofrequency energy was delivered under continuous EUS visualization, using a power setting of 30 W in continuous mode. One or more RFA applications were performed for each lesion, according to the endoscopist’s assessment. When required, energy was delivered at different sites within the same lesion to achieve complete coverage of the tumor. For multifocal disease, several lesions could be treated during the same endoscopic session using the same needle, with a change in needle trajectory for each target lesion, or during staged procedures depending on anatomy, procedure duration, and patient safety.

The number of applications, duration of energy delivery, number of needle passes, and treated tumor areas were adapted to tumor size, location, echogenic changes during ablation, and tissue impedance. Ablation was stopped in cases of marked impedance rise, satisfactory hyperechoic change within the lesion, technical difficulty, or safety concerns.

Intraprocedural vascular assessment was performed before puncture and immediately after completion of ablation whenever technically feasible. In all patients, this assessment included contrast-enhanced harmonic EUS after intravenous administration of sulfur hexafluoride microbubbles (SonoVue, Bracco International BV, Amsterdam, The Netherlands). Detective Flow Imaging (DFI; Arietta, Hitachi/Fujifilm Healthcare) and conventional Doppler were used as adjunctive non-contrast vascular imaging modalities to identify vessels in the puncture route and to assess residual vascular signal after ablation.

When residual intralesional enhancement or persistent DFI signal was observed after an RFA application, the electrode tip was repositioned, and additional energy delivery was considered, provided that the main pancreatic duct, major vessels, and gastrointestinal wall could be safely avoided. Conversely, the disappearance of enhancement/vascular signal within the target lesion was considered an intraprocedural endpoint supporting termination of the session.

### 2.5. Periprocedural Management

Periprocedural prophylaxis was performed according to the local institutional protocol. In the absence of contraindications, all patients received rectal non-steroidal anti-inflammatory drug prophylaxis (diclofenac 100 mg) and intravenous hydration with lactated Ringer’s solution for prevention of post-procedural pancreatitis. Hydration was individualized according to age, renal function, cardiovascular status, and fluid-overload risk. Antibiotic prophylaxis was not used routinely and was administered at the discretion of the treating physician.

Patients were monitored after the procedure for abdominal pain, fever, bleeding, pancreatitis, infection, perforation, and other procedure-related complications. Laboratory tests, including inflammatory markers, pancreatic enzymes, and liver tests, were obtained when clinically indicated.

### 2.6. Follow-Up and Response Assessment

Patients were followed after EUS-RFA using clinical assessment and contrast-enhanced imaging. Follow-up imaging was planned at 3, 6, and 12 months after the first RFA session, or earlier if clinically indicated. Contrast-enhanced CT in a pancreatic protocol was the preferred cross-sectional method for assessing ablation success for all lesions. Contrast-enhanced MRI was used when available. EUS with DFI and/or contrast-enhanced harmonic EUS was used as an adjunctive method when cross-sectional imaging was equivocal or when detailed local vascular assessment of the ablation zone was needed. Whenever possible, the same imaging modality was used at baseline and follow-up to improve comparability.

Tumor response was assessed on a per-lesion and per-patient basis. Complete response was defined as the absence of viable tumor within the treated lesion or a viable lesion without enhancement. Partial response was defined as a reduction in viable enhancing tumor tissue without complete disappearance. Stable disease was defined as the persistence of a viable tumor without significant progression. Progressive disease was defined as an increase in the size of the treated lesion, new enhancement within the ablation zone after previous response, or the appearance of new pancreatic or extra-pancreatic metastatic disease.

Residual viable tumor after initial RFA could be treated with additional EUS-RFA sessions if considered technically feasible and clinically appropriate, depending on response and multidisciplinary decision-making.

### 2.7. Outcomes

The primary endpoint was initial complete radiological response, defined as complete ablation of the treated pancreatic metastasis on first follow-up imaging, i.e., absence of viable enhancing tumor within the treated lesion. This endpoint was intentionally radiological and should not be interpreted as a clinical benefit or a survival advantage.

Secondary endpoints included technical success, adverse events, radiological response at 3, 6, and 12 months, need for repeat RFA, local progression, distant progression, progression-free survival, and overall survival.

Technical success was defined as successful placement of the RFA electrode within the target lesion and delivery of radiofrequency energy as planned. Adverse events were recorded during the procedure, during hospitalization or observation, and throughout follow-up. Early adverse events included bleeding, acute pancreatitis, perforation, infection, abdominal pain requiring admission or prolonged observation, and death. Late adverse events included pancreatic fistula, pseudocyst or fluid collection, main pancreatic duct stenosis, delayed pancreatitis, and delayed bleeding.

Adverse events were graded according to the ASGE lexicon and/or the AGREE classification for endoscopic adverse events [8,9].

### 2.8. Statistical Analysis

Descriptive statistics were used to summarize baseline characteristics, procedural details, and outcomes. Continuous variables were reported as the mean with standard deviation or the median with interquartile range, depending on the distribution. Categorical variables were reported as frequencies and percentages. Outcomes were analyzed per patient and, when appropriate, per lesion. Clinically relevant factors potentially associated with incomplete response or recurrence were assessed descriptively, including primary tumor type, lesion size, lesion location, number of pancreatic metastases, baseline enhancement pattern, proximity to the main pancreatic duct, number of RFA applications, total ablation time, and need for repeat treatment. Because of the limited cohort size, formal statistical testing of subgroup differences according to tumor location, lesion size, or primary tumor type was not performed; these factors were interpreted as hypothesis-generating or type was not performed; these factors were interpreted as hypothesis-generating.

## 3. Results

### 3.1. Patient and Lesion Characteristics

A total of 11 patients underwent EUS-guided radiofrequency ablation for pancreatic metastases during the study period. The mean age was 72.4 ± 6.4 years (median 72, range 58–81), and 7/11 patients (63.6%) were women. The median maximum treated lesion diameter was 10.5 mm (range 8–22 mm), including one multifocal case with two pancreatic metastases measuring 18 and 22 mm. The most common primary tumor was renal cell carcinoma (RCC), present in 7/11 patients (63.6%), followed by breast cancer in 2/11 (18.2%), melanoma in 1/11 (9.1%), and colorectal cancer in 1/11 (9.1%). All patients had local pancreatic metastases confirmed before treatment and were discussed in a multidisciplinary setting. Three patients in total (two with breast cancer and one with colorectal cancer) received an additional chemotherapy cycle. EUS-RFA was selected as a local treatment option when surgery was not feasible, was considered excessively invasive, or when an organ-preserving approach was preferred. Complete patient and lesion characteristics are presented in Table 1.

### 3.2. Procedural Characteristics

All procedures were performed using a 19G EUS-RFA needle electrode with a power setting of 30 W. The active tip length was 7 mm in 8/11 procedures (72.7%) and 10 mm in 3/11 (27.3%). The puncture route was transgastric in all recorded cases. Most lesions were treated with a single RFA application (8/11, 72.7%), while 2/11 (18.2%) required two applications and 1/11 (9.1%) required four applications during the same session. Median total RF application time was 35 s (range 25–87 s). Rectal NSAID prophylaxis and periprocedural hydration were used routinely, whereas prophylactic antibiotics were administered in 4/11 patients (36.4%). Prophylactic pancreatic duct stenting was not used in this cohort.

### 3.3. Technical Success

Technical success was achieved in 11/11 patients (100%). All patients had successful placement of the RFA electrode within the target lesion and delivery of radiofrequency energy as planned. Immediate post-treatment contrast-enhanced EUS/DFI assessment showed disappearance of enhancement in the treated area in all technically successful cases, suggesting complete immediate devascularization of the targeted lesion.

### 3.4. Early Radiological Response

The first follow-up imaging was performed at approximately three months. At the first follow-up, 10/11 patients (90.9%) showed initial complete radiological response, while 1/11 (9.1%) had partial ablation and underwent a second EUS-RFA session using 30 W, after which complete radiological response was confirmed.

### 3.5. Adverse Events

Adverse events occurred in 3/11 patients (27.3%). All were mild (AGREE grade I) and managed conservatively. Two patients developed mild acute pancreatitis, and one patient experienced post-procedural abdominal pain. No bleeding, perforation, pancreatic collection, main pancreatic duct stenosis, or procedure-related death was recorded. Median hospital stay was 4 days (range 3–16 days). Procedural technique and adverse events are presented in Table 2.

### 3.6. Follow-Up and Survival

**Figure 3:**
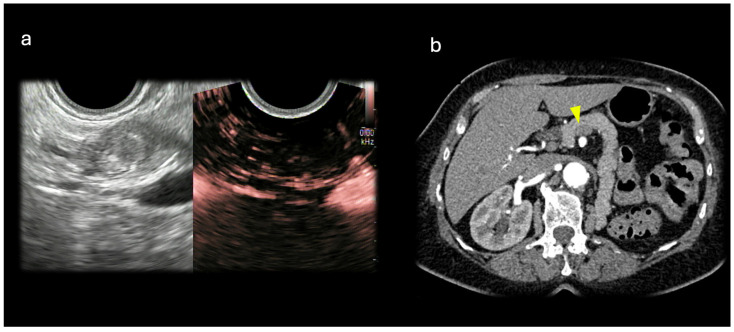
Representative post-ablation imaging assessment of the same treated lesion, demonstrating lack of residual vascular enhancement on EUS with DFI assessment (**a**) and on contrast-enhanced CT (**b**); yellow arrowhead.

Patient follow-up was performed according to protocol at 3, 6, and 12 months after the procedure; thereafter, patients were monitored in the outpatient setting for as long as data were available. The median follow-up from treatment to last available assessment was 338 days (range 22–1583 days). Initial complete radiological response was observed in 10/11 patients, and complete radiological response after one or two EUS-RFA sessions was observed in all cases (Figure 3). At last follow-up, 8/11 patients (72.7%) were alive, and 3/11 (27.3%) had died due to underlying malignancy. Follow-up and survival outcomes are presented in Table 3.

### 3.7. Exploratory Interpretation According to Lesion Size, Location, and Primary Tumor Type

Given the small sample size and the high overall local response rate, no formal comparisons were performed between pancreatic head and body/tail lesions, lesions <10 mm and ≥ 10 mm, or RCC and non-RCC metastases. Nevertheless, these variables were considered clinically relevant during procedural planning. Smaller lesions and lesions accessible through a safe transgastric or transduodenal trajectory may be technically more favorable. In contrast, lesions located close to the main pancreatic duct, major vessels, or the gastrointestinal wall may require shorter ablation applications, staged treatment, or a lower threshold for repeat ablation. Larger lesions may also require multiple applications and needle repositioning during the procedure. Additional technical challenges may arise when lesions are located far from the transducer or require access from the second portion of the duodenum, where scope angulation and stability can be limited. In the present cohort, complete response was ultimately achieved in all treated patients after one or two sessions; however, the small sample size precludes any conclusions regarding superior outcomes according to anatomical location, lesion size, or primary tumor subgroup.

## 4. Discussion

In this cohort of selected patients with metastatic lesions to the pancreas, EUS-guided radiofrequency ablation was technically feasible and associated with a high rate of local radiological response. Initial complete radiological response at first follow-up was observed in 10 of 11 patients, while the remaining patient achieved complete response after a second EUS-RFA session. The procedure was generally well tolerated, with only mild adverse events, including two cases of mild acute pancreatitis and one case of post-procedural abdominal pain, all managed conservatively. No bleeding, perforation, pancreatic fluid collection, main pancreatic duct stenosis, or procedure-related death was observed. These findings support the feasibility of EUS-RFA as an organ-preserving local treatment in carefully selected patients with pancreatic metastases, but they should not be interpreted as proof of oncological efficacy because of the small sample size, selection bias, and heterogeneity of primary tumor biology.

Metastatic involvement of the pancreas is rare and represents only a small proportion of pancreatic malignancies [3,4]. Among primary tumors metastasizing to the pancreas, clear-cell renal cell carcinoma is the most frequently reported [3,4]. Although RCC most commonly metastasizes to the lungs, bones, lymph nodes, and brain, it may also involve glandular organs, including the pancreas, thyroid, breast, and parathyroid glands. Interestingly, glandular metastases, particularly pancreatic metastases, have been associated with a more favorable prognosis compared with other metastatic sites [4,5,10]. This distinct biology may explain why local therapy is clinically relevant in selected patients, especially in the setting of isolated or oligometastatic disease.

Surgical resection remains the standard local treatment for selected patients with resectable pancreatic metastases. However, only a minority of patients present with a single, potentially resectable pancreatic lesion [3]. In addition, the survival benefit of metastasectomy has been most clearly observed for RCC metastases, while surgery for pancreatic metastases from other primary tumors is frequently performed with palliative intent [4]. Pancreatic surgery is technically demanding and may be associated with substantial morbidity, particularly in patients with small but multifocal lesions, lesions located in different pancreatic segments, previous abdominal surgery, advanced age, comorbidities, or limited functional reserve. Therefore, less invasive organ-preserving strategies are of increasing interest. In this context, EUS-RFA may represent a potential alternative or complementary local treatment, particularly when surgery is not feasible, carries excessive risk, or is not accepted by the patient.

Compared with previously published studies, our cohort has several clinically relevant differences. Published reports remain limited and heterogeneous, with variable sample sizes, tumor sizes, power settings, numbers of sessions, follow-up durations, and response definitions [5,6,7,10]. Reported complete response rates included 11.1% in the study by Figueiredo Ferreira et al., 30.4% in the series by Napoléon et al., 40% in the prospective study by Chanez et al., and 45.4% in the series by Stouvenot et al. [5,6,7,10]. In contrast, initial complete radiological response was observed in 90.9% of patients at first follow-up in our cohort, and complete radiological response was ultimately achieved in all treated patients after one additional session. This apparently higher response rate should be interpreted with substantial caution. It may partly reflect careful selection of small, well-visualized lesions, as the median lesion size in our study was 10.5 mm, which was smaller than in most previous reports, where mean tumor size ranged from approximately 14.4 mm to 34 mm [5,6,7,11]. The present results should therefore be viewed as hypothesis-generating rather than comparative evidence of superiority.

Another advantage of the present study is the systematic procedural characterization. In previous reports, the number of applications, application time, active tip length, and intraprocedural response assessment were often variable or incompletely reported [5,6,7,10]. In our cohort, all procedures were performed using a 19G EUS-RFA needle with internal cooling and a power setting of 30 W. The active tip length, number of applications, total ablation time, puncture route, use of contrast assessment, and immediate post-treatment vascular response were recorded. Most lesions were treated with a single RFA application, and the median total RF application time was 35 s. Only one patient required a second EUS-RFA session, after which a complete response was achieved.

From a practical perspective, tumor location and size are likely to influence EUS-RFA outcomes mainly through technical feasibility and safety, rather than through oncological biology alone. Lesions in the pancreatic head or uncinate process may be accessed from the duodenum and may lie close to the common bile duct, the main pancreatic duct, and vascular structures, potentially increasing technical complexity and the risk of pancreatitis or ductal injury. Lesions in the pancreatic body or tail are often accessible through a transgastric route, which may facilitate needle stability; however, tail lesions may still be challenging if they are small, deeply located, or adjacent to splenic vessels. Similarly, lesions <10 mm may be easier to ablate completely but may be more difficult to target precisely, whereas lesions ≥10 mm may require multiple applications, a longer cumulative ablation time, repositioning of the electrode, or staged treatment. These considerations support individualized planning rather than a uniform protocol (Table 4).

The present study also differs from the existing literature by including not only RCC metastases but also pancreatic metastases from breast cancer, melanoma, and colorectal cancer. This is important because pancreatic metastases should not be regarded as a homogeneous therapeutic target. Metastatic lesions may differ substantially according to the primary tumor in terms of vascularity, stromal composition, growth pattern, tumor capsule, necrotic component, and sensitivity to thermal injury [4,11,12]. Hypervascular RCC metastases may be particularly suitable for contrast-enhanced EUS- or DFI-guided devascularization, whereas metastases from colorectal cancer, breast cancer, melanoma, lung cancer, or other primaries may have different enhancement patterns and may require different ablation strategies. Therefore, although a complete response to treatment was observed in our study, a single universal EUS-RFA protocol for all pancreatic metastases is unlikely to be appropriate.

The biological heterogeneity of pancreatic metastases may also have direct implications for the expected response to thermal ablation. In contrast to primary pancreatic ductal adenocarcinoma, pancreatic metastases represent a group of biologically distinct tumors that retain, at least in part, features of their primary malignancy. This may influence both heat delivery and post-ablation tumor behavior. Relevant factors include tumor vascularity and perfusion, stromal density, cellular architecture, immune microenvironment, baseline necrosis, and the ability of residual cells to activate heat-stress survival pathways [11,12,13,14,15]. Therefore, differences in local response after EUS-RFA should not be interpreted only as a technical phenomenon, but may also reflect tumor-specific biology.

This concept is particularly relevant for RCC. Molecular and immunogenomic studies have shown that RCC metastases to the pancreas constitute a distinct metastatic phenotype characterized by relatively indolent biology, heightened angiogenesis, and an uninflamed stromal microenvironment [11]. These features may explain the favorable prognosis often observed in patients with pancreatic RCC metastases and support the rationale for focal local therapies in selected oligometastatic patients. At the same time, the marked hypervascularity of RCC metastases may theoretically create a dual effect during RFA: it allows precise vascular imaging and immediate assessment of devascularization using CE-EUS or DFI, but it may also increase convective cooling and heat-sink effects near vessels, potentially reducing complete thermal injury at the tumor margins [11,12,13].

The response of non-RCC pancreatic metastases may be different. Breast cancer, colorectal cancer, melanoma, lung cancer, and sarcoma metastases may vary in stromal content, enhancement pattern, growth kinetics, immune contexture, and propensity for systemic progression. Moreover, experimental and translational studies in other tumor models suggest that insufficient or sublethal thermal injury may promote survival of residual tumor cells through mechanisms such as heat-shock protein expression, hypoxia-related signaling, autophagy, epithelial–mesenchymal transition, and increased invasive potential [14,15]. Although these mechanisms have not been specifically validated for EUS-RFA of pancreatic metastases, they provide a biologically plausible explanation for why different metastatic primaries may require different ablation margins, energy settings, imaging endpoints, or repeat treatment strategies. Future studies should therefore report outcomes separately according to primary tumor type and should integrate vascular imaging, lesion biology, and radiological response criteria into treatment algorithms.

A key unresolved issue is the lack of standardized RFA parameters for pancreatic metastases. Most pancreatic EUS-RFA experience has been generated in pancreatic neuroendocrine tumors and selected cystic pancreatic lesions, and these settings cannot be automatically extrapolated to metastatic tumors [1,2]. Published studies of pancreatic metastases have used variable power settings, most commonly 50 W, with different application times and numbers of applications per lesion [5,6,7]. In contrast, our cohort used 30 W, with treatment individualized according to lesion size, location, vascularity, EUS appearance, tissue impedance, and safety considerations. This approach may be relevant because lower wattage applied for a longer time has been suggested to produce slower, but more controlled, thermal injury through a thermal diffusivity effect. Conversely, higher power settings may lead to rapid charring around the needle, increased impedance, and reduced current conduction, potentially resulting in smaller ablation zones [16].

The outcomes of EUS-RFA for pancreatic metastases should also be distinguished from those reported for conventional pancreatic ductal adenocarcinoma [17]. In metastatic pancreatic lesions, EUS-RFA is typically directed at small, well-demarcated targets in patients with controlled or oligometastatic disease, with the procedural endpoint being local devascularization or complete ablation of a discrete lesion. By contrast, EUS-RFA in pancreatic ductal adenocarcinoma has mainly been investigated as an adjunctive or palliative approach in locally advanced, infiltrative, hypovascular, and desmoplastic tumors, often with close involvement of the pancreatic duct, bile duct, and peripancreatic vessels. Therefore, response rates, safety profiles, and clinical endpoints cannot be directly extrapolated between pancreatic metastases and conventional pancreatic cancer. For pancreatic ductal adenocarcinoma, local ablation is unlikely to replace systemic therapy and is generally evaluated in terms of local cytoreduction, symptom control, feasibility, and survival, rather than complete ablation of a small metastatic focus.

Another important technical element in our study was the use of Doppler, contrast-enhanced EUS, and/or DFI before and after ablation. All examined lesions showed a hypervascular pattern before treatment, and complete disappearance of enhancement was observed after ablation. This suggests that immediate devascularization may be a useful intraprocedural endpoint during EUS-RFA of pancreatic metastases. Size-based assessment alone may be insufficient early after ablation, because the treated lesion may persist morphologically despite loss of viable enhancing tissue. Functional vascular imaging may therefore help determine whether additional applications are needed during the same session and may be especially useful for hypervascular metastases.

The need for a second EUS-RFA session in one patient also supports the concept that EUS-RFA for pancreatic metastases should not necessarily be viewed as a single-session therapy. For some lesions, particularly those close to the pancreatic duct, major vessels, or the gastrointestinal wall, staged ablation may be safer than aggressive single-session treatment. Repeatability is one of the potential advantages of EUS-RFA compared with surgery, as it allows treatment to be adapted over time and may be useful in multifocal pancreatic disease or in patients requiring parenchyma-preserving local control.

The clinical role of EUS-RFA should be interpreted within a broader oncological strategy. Complete local ablation of a pancreatic metastasis does not necessarily translate into improved progression-free or overall survival, especially in patients with active extra-pancreatic disease. Therefore, EUS-RFA should not be considered a replacement for systemic therapy when systemic treatment is indicated. Rather, it may serve as a local treatment tool in carefully selected patients with isolated or oligometastatic pancreatic disease, small progressive lesions in otherwise controlled systemic disease, or patients in whom surgery would be disproportionate to the disease burden. In our cohort, 3 of 11 patients received additional chemotherapy after EUS-RFA, supporting the concept that local ablation may be integrated into a wider individualized oncological strategy rather than used in isolation.

The safety profile observed in this cohort was encouraging but should be interpreted cautiously. Mild acute pancreatitis occurred in two patients, which remains an expected adverse event after thermal ablation within the pancreas. This highlights the importance of careful pre-procedural planning, avoidance of the main pancreatic duct when possible, Doppler assessment to avoid vessels, and consideration of prophylactic measures such as rectal non-steroidal anti-inflammatory drugs and intravenous hydration. In our cohort, pancreatitis prophylaxis and hydration were used in all patients, and no prophylactic pancreatic duct stenting was performed. No delayed ductal stenosis or pancreatic fluid collection was observed, although the small sample size limits conclusions regarding uncommon delayed complications.

Our findings and the available literature also support several methodological priorities for future standardization of EUS-RFA for pancreatic metastases. Future protocols could be focused on defining power settings, application duration, impedance-based stopping criteria, criteria for repeat or staged ablation, and the role of vascular imaging according to the baseline enhancement phenotype of each metastasis. Real-time control should integrate B-mode visualization of the hyperechoic ablation zone, tissue impedance, Doppler/DFI assessment, and, when available, contrast-enhanced harmonic EUS; however, the expected value of each endpoint may differ between hypervascular RCC metastases and less vascular or heterogeneously enhancing metastases from other primaries. Prospective multicenter studies should therefore standardize not only cross-sectional CT/MRI response criteria and follow-up time points, but also the reporting of lesion size, location, primary tumor type, enhancement pattern, intraprocedural vascular endpoint, and need for repeat treatment.

This study has several limitations. First, the cohort is small, reflecting the rarity of pancreatic metastases treated with EUS-RFA, and this limits the precision of efficacy and safety estimates. Second, although the inclusion of different primary tumors is one of the novel aspects of the study, it also introduces heterogeneity. Metastases from RCC, breast cancer, melanoma, and colorectal cancer differ in biology, vascularity, growth kinetics, systemic treatment options, and prognosis [4,11,17]. Third, response criteria after EUS-RFA for pancreatic metastases are not standardized, and published studies use different definitions of complete response, partial response, and non-response. Finally, the small number of patients precluded meaningful analysis of predictors of response, recurrence, or adverse events.

An additional methodological limitation is that DFI and contrast-enhanced EUS cannot be completely interpreted in an identical manner for all metastatic tumor types. The majority of lesions in this cohort were hypervascular, and RCC metastases are particularly suitable for intraprocedural vascular assessment. In contrast, breast cancer, colorectal cancer, melanoma, and other metastases may show variable, moderate, hypovascular, heterogeneous, or rim-enhancing patterns on contrast-enhanced imaging. Consequently, immediate disappearance of the DFI/CE-EUS signal may be a more robust endpoint in hypervascular lesions than in hypovascular or heterogeneously enhancing lesions. For these tumors, treatment success should rely primarily on standardized contrast-enhanced CT or MRI follow-up, supported by EUS findings when appropriate. This limitation restricts the generalizability of a CE-EUS/DFI-guided devascularization endpoint across all pancreatic metastases and should be specifically addressed in future studies stratified by primary tumor type and enhancement phenotype [17,18,19,20,21].

## 5. Conclusions

In conclusion, EUS-guided radiofrequency ablation appears to be a feasible and generally well-tolerated organ-preserving local treatment for selected patients with pancreatic metastases. The technique may be particularly useful for small lesions when surgery is not feasible, carries excessive risk, or is not preferred. However, given the limited sample size, selection bias, heterogeneity of metastatic tumor biology, and lack of standardized ablation parameters, these results should be considered preliminary and hypothesis-generating. Larger prospective multicenter studies with uniform RFA protocols, standardized imaging response criteria, longer follow-up, and clinically meaningful oncological endpoints are needed to define the role of EUS-RFA in the treatment algorithm for metastatic lesions to the pancreas.

## Figures and Tables

**Table 1:** Patient and lesion characteristics.

Variable	Value
Patients, *n*	11
Age, years, mean ± SD	72.4 ± 6.4
Age, years, median (range)	72 (58–81)
Female sex	7/11 (63.6%)
Male sex	4/11 (36.4%)
Charlson Comorbidity Index, median (range)	5 (3–6)
ASA class, median (range)	2 (1–3)
BMI, kg/m^2^, median (range)	24.0 (17.7–30.1)
Current/past smoker	7/11 (63.6%)
Primary tumor:	
renal cell carcinoma	7/11 (63.6%)
breast cancer	2/11 (18.2%)
melanoma	1/11 (9.1%)
colorectal cancer	1/11 (9.1%)
Maximum treated lesion diameter, mm, median (range)	10.5 (8–22)
Multifocal pancreatic disease	1 patient (two lesions: 18 and 22 mm)
MPD caliber	Normal in 11/11 (100%)

**Table 2:** Procedural technique and adverse events.

Variable	Value
EUS-RFA procedures, *n*	11
Pancreatitis prophylaxis	11/11 (100%)
Hydration	11/11 (100%)
Antibiotic prophylaxis	4/11 (36.4%)
Prophylactic ERCP with pancreatic stent placement	0/11 (0%)
Vessel proximity	1/11 (9.1%)
Technical success	11/11 (100%)
Exposed active tip:	
7 mm	8/11 (72.7%)
10 mm	3/11 (27.3%)
Power setting	30 W
Number of RFA applications:	
1	8/11 (72.7%)
2	2/11 (18.2%)
4	1/11 (9.1%)
Total RF application time, seconds, median (range)	35 (25–87)
Puncture route	Transgastric
Any adverse event	3/11 (27.3%)
Type of adverse event	
Acute pancreatitis	2/11 (18.2%)
Abdominal pain	1/11 (9.1%)
Timing of adverse events	<14 days in 3/11 (27.3%)
AE severity	AGREE grade I in 3/11 (27.3%)
Medical intervention for AE	Conservative treatment in 3/11 (27.3%)
Hospital stay, days, median (range)	4 (3–16)
Bleeding/perforation/pancreatic collection/MPD stenosis/procedure-related death	0/11 (0%)

**Table 3:** Follow-up and survival outcomes.

Variable	Value
Initial complete radiological response at first follow-up	10/11 (90.9%)
Partial radiological response at first follow-up	1/11 (9.1%)
Second EUS-RFA session	1/11 (9.1%)
Adverse event after the second treatment	0/1 (0%)
Complete radiological response after the second treatment	1/1 (100%)
Therapy after EUS-RFA: none	8/11 (72.7%)
Therapy after EUS-RFA: chemotherapy	3/11 (27.3%)
Median follow-up from treatment to last available assessment, days	338
Follow-up range, days	22–1583
Alive at last follow-up	8/11 (72.7%)
Dead at last follow-up	3/11 (27.3%)
Cause of death	Underlying malignancy

**Table 4:** Potential factors that may influence EUS-RFA outcomes in pancreatic metastases.

Factor	Potential Influence	Practical Implication
Location	Head/uncinate lesions may be closer to the bile duct, MPD, and vessels; body/tail lesions often allow transgastric access but may be close to splenic vessels.	Plan access route and ductal/vascular safety; consider staged or conservative ablation near critical structures.
Size	<10 mm lesions may be easier to ablate but harder to target; lesions ≥10 mm may require more applications, longer ablation time, or repeat treatment.	Report size-based outcomes separately; avoid overaggressive single-session ablation in larger or anatomically risky lesions.
Primary tumor	RCC lesions are often hypervascular and suitable for DFI/CE-EUS-guided devascularization; non-RCC metastases may have different vascularity and biology.	Stratify future outcomes by primary tumor type rather than pooling all pancreatic metastases.
Vascularity/vessels	Vascular imaging enables real-time devascularization assessment, while adjacent vessels may cause heat-sink effects.	Use Doppler, DFI, or CE-EUS before and after ablation as an intraprocedural supportive endpoint whenever feasible.

## Data Availability

The data underlying this article are available from the corresponding author upon reasonable request, subject to institutional and ethical restrictions.
